# Patient and staff perspective toward marker‐less patient setup accuracy in breast radiotherapy

**DOI:** 10.1002/acm2.70452

**Published:** 2026-01-02

**Authors:** Puntiwa Oonsiri, Sornjarod Oonsiri, Sakda Kingkaew, Mananchaya Vimolnoch, Jumnong Kumkhwao, Photong Duangsuphan, Panicha Nualsutha, Nattawat Samranjai, Siriporn Wong, Kitwadee Saksornchai

**Affiliations:** ^1^ Division of Radiation Oncology, Department of Radiology King Chulalongkorn Memorial Hospital, The Thai Red Cross Society Bangkok Thailand; ^2^ Division of Radiation Oncology Department of Radiology Faculty of Medicine Chulalongkorn University Bangkok Thailand

**Keywords:** breast cancer, marker‐less, surface‐guided radiotherapy

## Abstract

**Purpose:**

This study comprised two cohorts. The first assessed patient and radiation therapist (RTT) perspectives on temporary ink skin markings. The second evaluated whether integrating a six‐degree‐of‐freedom (6D) couch could further improve setup accuracy, setup time, IGRT time, and total treatment time when used with marker‐less or marker‐based workflows.

**Methods:**

Questionnaires were completed by 72 breast cancer patients and 29 radiation therapists (RTTs) using a 6‐point Likert scale (0 = never to 5 = always). The questionnaire assessed emotional experiences related to temporary skin markings and the level of interest in a marker‐less approach. Forty patients were assigned to four setup groups: markers without 6D couch (M), markers with 6D couch (M_6D), marker‐less without 6D couch (ML), and marker‐less with 6D couch (ML_6D). Setup accuracy, setup time, image‐guided radiotherapy (IGRT) time, and total treatment time were compared.

**Results:**

About 90% of both patients and RTTs preferred a marker‐less setup. Marker‐less techniques combined with 6D couch achieved comparable positioning accuracy to marker‐based methods. Maximum mean setup deviations in the longitudinal axis were 3.3 ± 1.3 mm (M), 3.3 ± 1.5 mm (M_6D), 2.8 ± 1.3 mm (ML), and 3.0 ± 1.5 mm (ML_6D). No significant differences were found in setup or IGRT time. Marker‐less setups reduced total treatment time by 27.8%.

**Conclusion:**

Marker‐less setups using SGRT are feasible for breast radiotherapy, providing comparable accuracy and reduced treatment times. Both patients and RTTs preferred this method over traditional marker‐based approaches.

## 1 INTRODUCTION

Skin markings, either invasive (tattoos) or non‐invasive (temporary ink), have long been used to assist patient setup in radiotherapy. These markings are typically used in conjunction with laser alignment to ensure consistent patient positioning and treatment accuracy.^1^ In breast cancer treatment, marks are usually placed on the anterior mid‐sternum, lateral chest wall, field borders, and at the isocenter, depending on institutional protocol. When regional lymph nodes are included, markings may extend to more exposed anatomical areas, potentially causing discomfort and psychological distress by serving as a constant reminder of the disease.^2^, ^3^

Surface‐guided radiotherapy (SGRT) enhances real‐time patient positioning, particularly during deep inspiration breath hold (DIBH), and may reduce or eliminate the need for skin markings, thereby improving patient comfort and workflow efficiency.^4^ SGRT uses non‐ionizing visible light to capture a real‐time three‐dimensional (3D) surface using multiple camera pods. The reference surface, typically generated from simulation, is compared with the real‐time surface to guide patient setup with six degrees of freedom (6DOF), including both translational and rotational adjustments. Although the primary clinical application of SGRT has been monitoring patient breath‐hold during treatment,^3^ patient setup in many centers still relies on skin markings. These marks can fade over time and often require reapplication, which may increase patient concerns and add to the clinical workload.

Previous studies have shown that SGRT improves setup accuracy and may positively influence patient emotional experience.^1^, ^2^, ^3^, ^4^ However, few investigations have evaluated the emotional impact of skin markings from both patient and radiation therapist (RTT) perspectives. In this study, we used patient and RTT questionnaires to assess these experiences. Additionally, we examined whether integrating SGRT with a six‐degree‐of‐freedom (6D) couch provides further improvements in setup accuracy compared with conventional workflows. The outcomes evaluated included setup accuracy, setup time, image‐guided radiotherapy (IGRT) time, and total treatment time.

## 2 MATERIALS AND METHODS

### 2.1 Skin marking questionnaire

The first cohort in our study included 72 breast cancer patients who underwent radiotherapy with temporary skin markings between January 2025 and March 2025 and completed a questionnaire during the final week of their treatment. Patients were categorized into two groups based on employment status: employed and retired/unemployed. Employed patients may experience greater concern regarding the visibility of temporary skin markings due to professional dress codes, whereas unemployed patients may have fewer social exposures and thus different emotional responses. The questionnaire included three sections: (1) General information, (2) Emotional experiences related to temporary skin markings, and (3) interest in a marker‐less approach. It comprised eight items and required approximately 3 min to complete. Each item used a 6‐point Likert scale ranging from never (score = 0) to always (score = 5), with higher scores reflecting greater emotional impact or discomfort associated with temporary skin markings. Section scores were converted into percentages for analysis.

The questionnaire was also administered to all 29 RTTs to assess their perspectives on patients’ concerns related to temporary skin markings. RTTs were asked to rate how frequently they observed or were informed about patients’ concerns, such as anxiety, hygiene issues, fading of the marker, repeated redraw, or allergic reactions. Responses were recorded using the same 6‐point Likert scale (0 = never to 5 = always), with higher scores indicating more frequent encounters with patient distress or concern. Section scores were likewise converted to percentages for analysis. RTTs were categorized into junior and senior groups based on their experience in breast positioning, using 5 years as the threshold. This distinction was made because greater clinical experience may influence sensitivity to patients’ emotional responses. The study received approval from the Institutional Review Board.

### 2.2 Patient simulation and planning

A distinct cohort of patients, separate from those who completed the questionnaire, was assigned to either the ‘marker’ or ‘marker‐less’ group. Immobilization was achieved using a Vac‐Lok device with knee support (CIVCO Medical Solutions, Iowa, USA), with both arms positioned above the head. CT simulation was performed using a GE Revolution scanner (GE Medical Systems, Madison, USA). Patients with left‐sided breast cancer who were eligible for deep inspiration breath hold (DIBH) underwent respiratory coaching prior to simulation.^5^ Setup fields, including the anterior and lateral borders of the ipsilateral breast, were generated by a radiation oncologist using the AdvantageSim MD virtual simulation system (GE Medical Systems).

**FIGURE 1:**
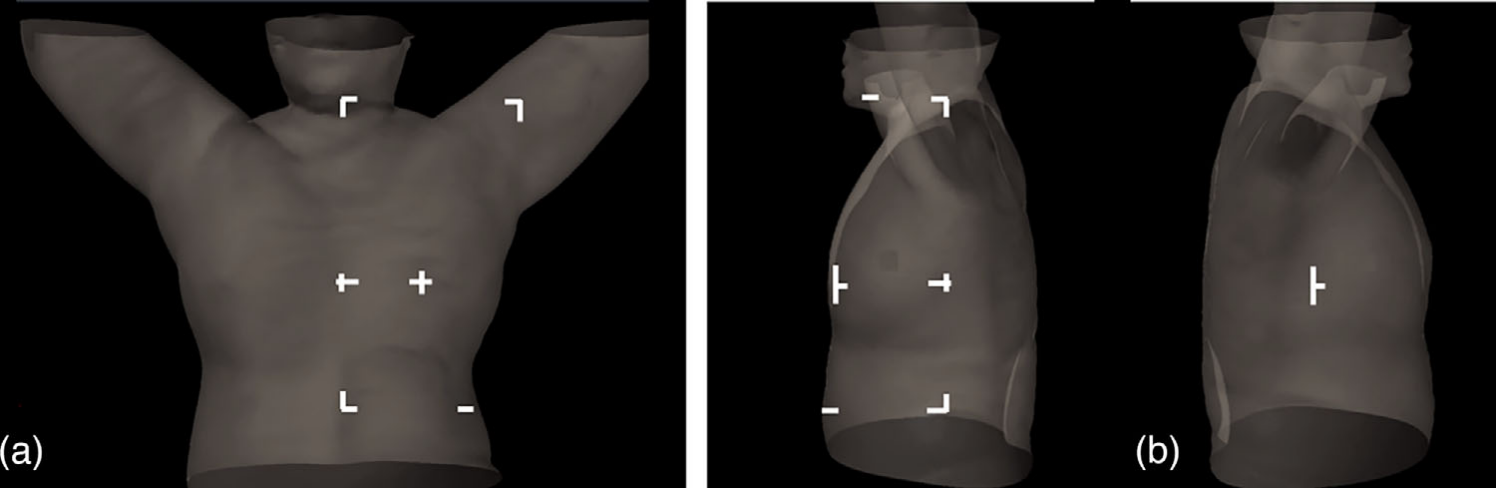
Temporary skin markings of the setup field: (a) anterior view, and (b) lateral view.

In the marker group, temporary skin markers using gentian violet dye were applied. Reference lines were drawn on the patient's skin at the midline of the chest, both lateral mid‐thorax regions, the upper corners approximately 13–15 cm superior to the isocenter, the isocenter itself, and the lower corners. For patients receiving regional nodal irradiation, the upper corner marking could facilitate head alignment and ensure proper positioning. The locations of the temporary skin markings are shown in Figure 1. In contrast, patients in the marker‐less group had no skin marking applied. Instead, a reference line corresponding to the longitudinal laser at the isocenter of the setup field was drawn on the Vac‐Lok immobilization device. Once the patient was positioned, this line was aligned with the room lasers to achieve a reproducible setup close to the treatment isocenter.

Patients treated with the volumetric modulated arc therapy (VMAT) technique were included in this study. VMAT planning was performed in Eclipse v16.1 (Varian Medical Systems, Palo Alto, CA) using the same isocenter established during CT simulation,^6^, ^7^ with a prescribed dose of 42.4 Gy in 16 fractions. To ensure consistency in beam‐on parameters, VMAT plans consisted of four partial arcs, with gantry angles of 240°–50° and 135°–310° for the right and left breasts, respectively. Because of the limited maximum multileaf collimator (MLC) leaf span in Varian linear accelerators, the X‐direction jaw width is restricted to 16 cm^6^. To improve dose coverage of the planning target volume (PTV), the collimator was rotated to 90° for two arcs, allowing adequate coverage of the superior and inferior portions of the PTV.

Treatments were delivered using four TrueBeam linear accelerators (Varian Medical Systems, Palo Alto, CA), two of which were equipped with 6D couches. Surface guidance was performed using AlignRT (Vision RT, London, UK), which uses ceiling‐mounted 3D cameras for real‐time surface imaging and motion monitoring. The treatment plan and the patient's body contour were exported from the treatment planning system to AlignRT.

### 2.3 Setup accuracy

Our study included a total of 200 treatment fractions (40 patients × 5 fractions per patient), which were divided into four groups: 10 patients with markers and without 6D couch (M), 10 patients with markers and a 6D couch (M_6D), 10 patients marker‐less and without a 6D couch (ML), and 10 patients marker‐less and with a 6D couch (ML_6D). Each group consisted of 5 patients receiving free‐breathing treatment (right breast) and DIBH treatment (left breast).

For patients in marker groups, positioning was performed by aligning the temporary skin markings placed during the CT simulation with the room lasers and the light‐field borders of the setup fields. IGRT was performed during the first three fractions and subsequently on a weekly basis, in accordance with our institutional protocol. Cone‐beam computed tomography (CBCT) was used for IGRT, beginning with automatic registration followed by verification and manual adjustment by experienced RTT as needed. Translational couch shifts were evaluated in the lateral (Lat), longitudinal (Long), and vertical (Ver) directions. For patients treated using a 6D couch, rotational errors in yaw, pitch, and roll were also assessed. Couch shifts were applied prior to beam delivery. If the online‐match couch shift exceeded 5 mm or 3°, the CBCT was repeated following the couch adjustment. Temporary skin marking typically fades within a few days due to skin exfoliation, hygiene practices, and friction from clothing. As a result, marking is frequently required during multi‐fraction treatments or when markings are unintentionally removed. RTTs must manually reapply the markings, contributing to prolonged treatment times.

**FIGURE 2:**
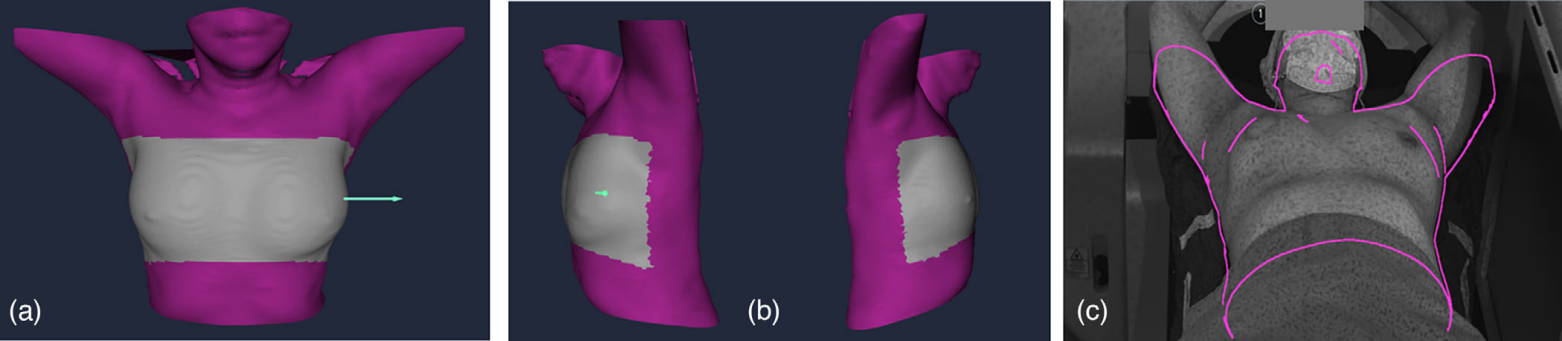
Regions of interest for the 3D body surface in AlignRT: (a) anterior view, (b) lateral view, and (c) video‐postural display.

For patients in the marker‐less group, the reference point of the longitudinal laser drawn on the Vac‐Lok device was aligned with the wall‐mounted room lasers. Patient positioning was performed using AlignRT through the video‐postural module, which displays an outline of the reference surface to detect initial misalignments. The imported body contour from treatment planning was then matched with the real‐time surface using a defined region of interest (ROI). The ROI encompassed both breasts and extended laterally to the midaxillary line to reduce camera occlusion caused by the gantry, kV x‐ray source, and rotating detector.^8^, ^9^ The ROI used for surface matching and the video‐postural display are shown in Figure 2. AlignRT setup tolerances were 3 mm for translational deviations and 3° for rotational deviations. CBCT was performed for position verification following the same protocol used in the marker‐based groups.

**FIGURE 3:**
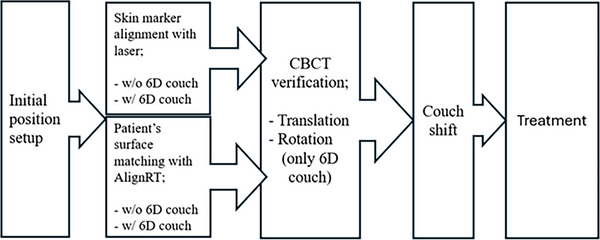
Treatment workflow.

To complement this study, the setup time, defined as the interval from the moment the patient lay down on the couch to the moment they were ready for imaging, was recorded. Additionally, the IGRT time, defined as the duration from image acquisition to completion of all position adjustments, and total treatment time was defined as the interval from patient entry to exit of the treatment room. These metrics were used to assess workflow efficiency for each setup technique. The daily treatment workflow is shown in Figure 3.

### 2.4 Statistical analysis part

For Likert scale responses, mean scores were calculated, and comparisons were performed using independent t‐tests between employed and retired/unemployed patients, and between junior and senior RTTs. Setup accuracy is reported as mean ± standard deviation. The Shapiro–Wilk test was used to assess the data normality of the continuous variable. Generalized estimating equations (GEE) with a linear model and an exchangeable working correlation structure were applied to compare repeated‐measure outcomes among the four groups. All *p*‐values were two‐sided, and statistical significance was defined as *p* < 0.05. Statistical analyses were conducted using Stata version 18.5 (StataCorp, College Station, TX, USA)

## 3 RESULTS

### 3.1 Skin marking questionnaire

In the first cohort, a total of 72 patient questionnaires regarding temporary skin markers were completed and analyzed. The median age was 50 years (range, 29–77 years) in the employed group and 63 years (range, 24–77 years) in the retired/unemployed group. Among the 29 RTT respondents (7 males, 22 females) questionnaires, the median age was 26 years (range, 25–28 years) for junior staff and 33 years (range, 29–59 years) for senior staff.

**FIGURE 4:**
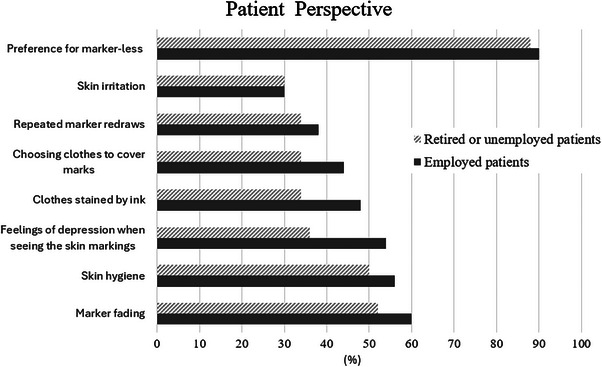
Patients’ emotional experiences related to temporary skin markings.

**FIGURE 5:**
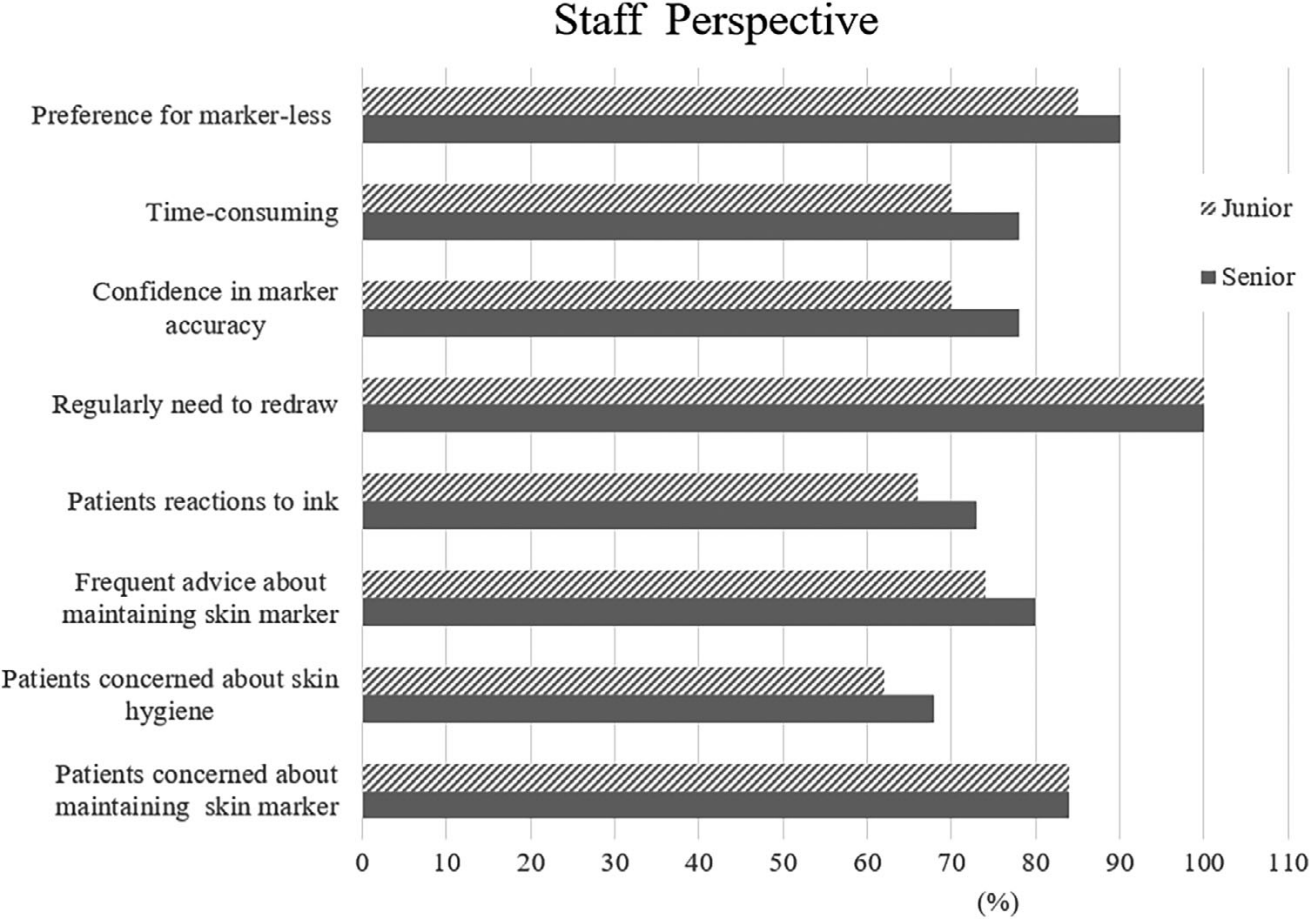
Radiation therapists’ observations of patients’ concerns regarding temporary skin markings.

Figure 4 illustrates the emotional experiences associated with temporary skin markings in employed and unemployed patients. Both groups demonstrated similarly high interest in a marker‐less approach, with nearly 90% expressing preference; no statistically significant difference was observed (*p* = 0.58). However, employed patients reported significantly higher stress levels (*p* < 0.05) related to marker redraws, difficulty choosing clothing to conceal marks, ink stains, a feeling of depression when seeing the markings, skin hygiene concerns, and marker fading. No significant difference in skin irritation was found between the two groups (*p* = 0.23). Among RTTs, 100% reported needing to redraw temporary skin markers during treatment to maintain accurate setup positioning. Approximately 90% expressed interest in a marker‐less setup workflow. Additionally, about 80% of RTTs frequently addressed patient concerns regarding marker fading, and 68% reported providing guidance on skin hygiene. All RTTs in both junior and senior groups indicated that marker redraws were routinely required due to fading. No statistically significant differences were observed between junior and senior RTTs across any questionnaire items. The experiences of RTTs regarding temporary skin marking are presented in Figure 5.

### 3.2 Setup accuracy

**FIGURE 6:**
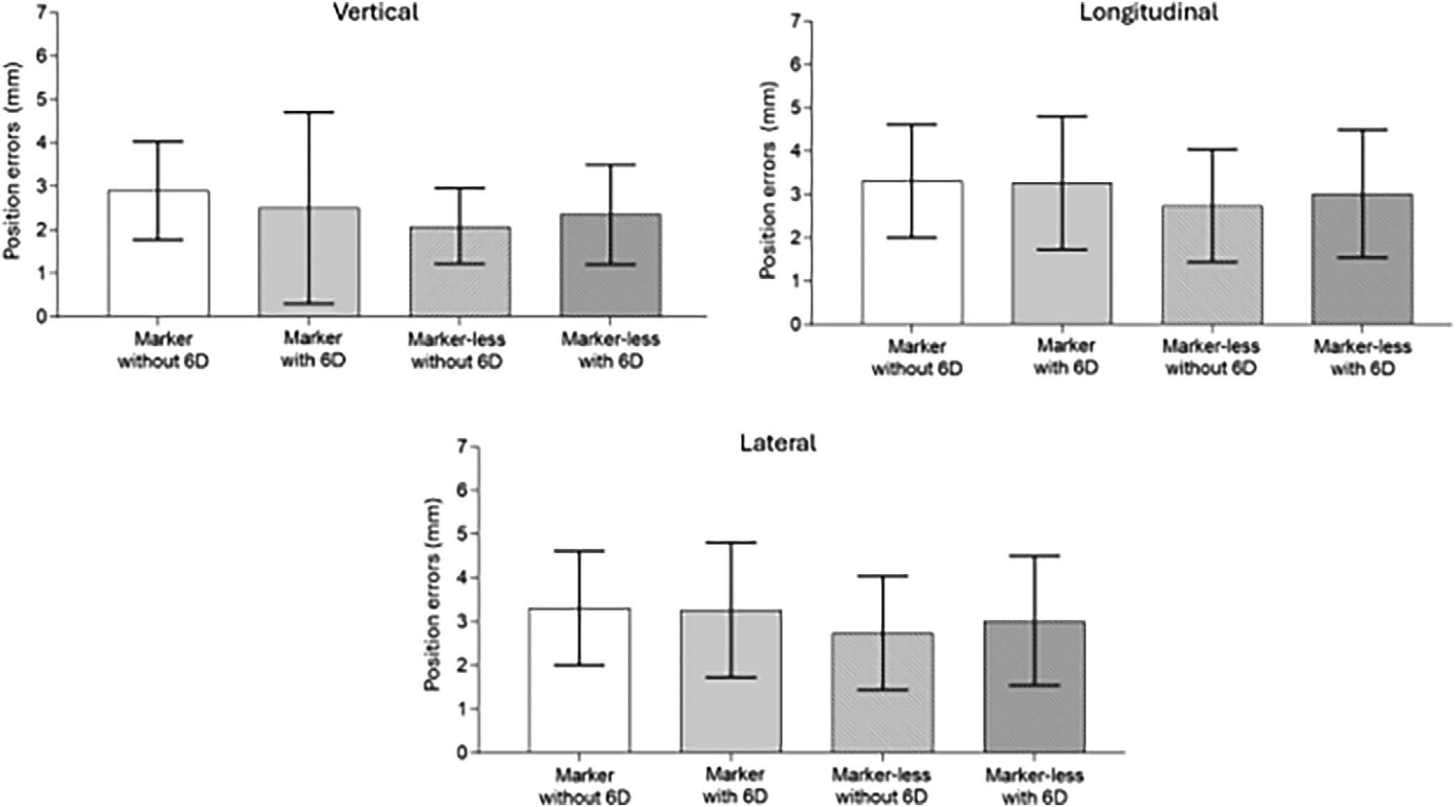
Bar graph comparing translational setup errors (vertical, longitudinal, and lateral) with and without a 6D couch.

**FIGURE 7:**
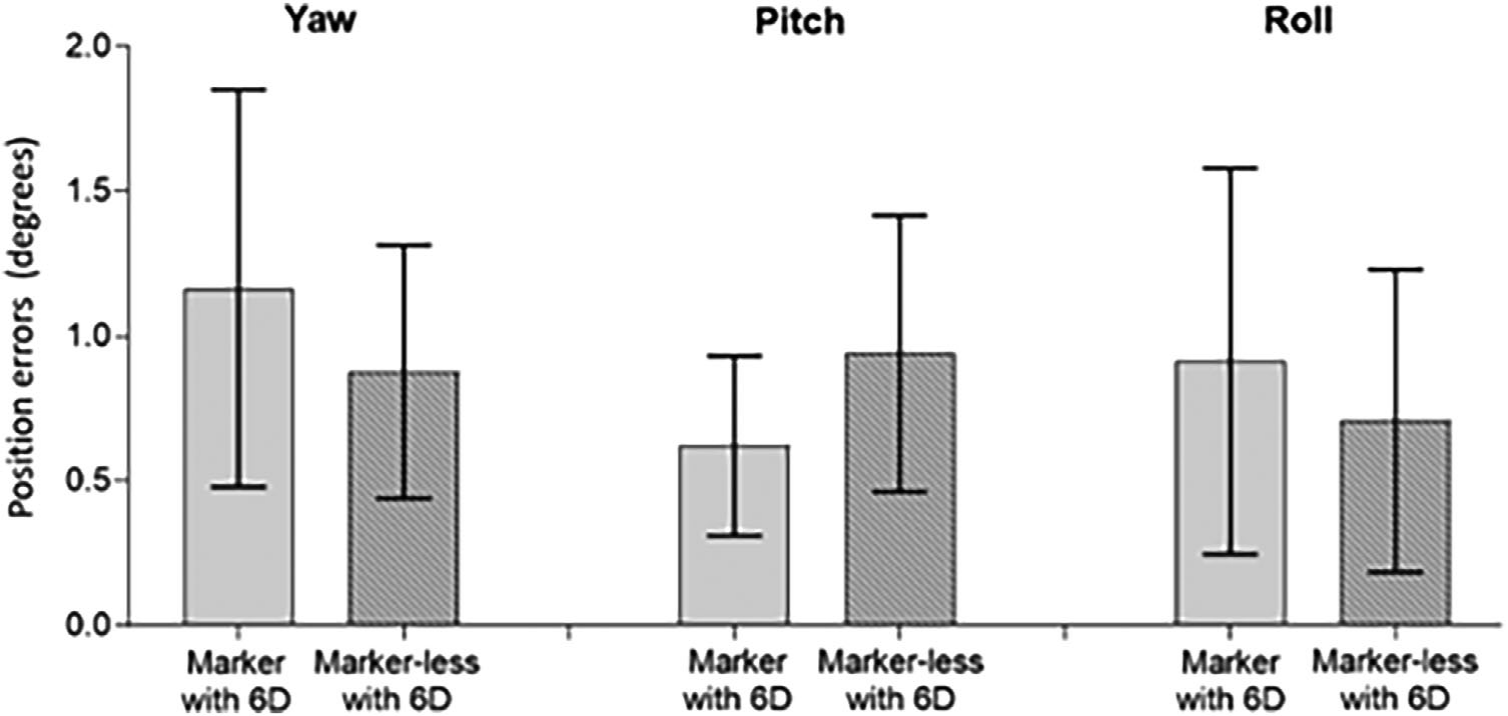
Bar graph comparing rotational setup errors (yaw, roll, and pitch) between marker and marker‐less groups.

In the second cohort, each patient was assigned to one of four groups based on setup method and couch type. The average translational and rotational couch shifts for all groups are presented in Figures 6 and 7, respectively. The results of the GEE analysis comparingsetup accuracy among four groups are summarized in Table 1. Overall, the marker‐less technique combined with a 6D couch demonstrated positioning accuracy comparable to that of marker‐based methods. No statistically significant differences were observed in translational or rotational couch shifts among the groups, except for a difference between the M and ML groups in the lateral direction (*p* = 0.02). Translational errors were greatest in the longitudinal direction, followed by vertical and lateral directions. The maximum average longitudinal deviations were 3.3 ± 1.3 mm, 3.3 ± 1.5 mm, 2.8 ± 1.3 mm, and 3.0 ± 1.5 mm for the M, M_6D, ML, and ML_6D groups, respectively.

**TABLE 1:** GEE analysis of setup accuracy comparisons among four groups: Marker without 6D couch (M), marker with 6D couch (M_6D), marker‐less without 6D couch (ML), and marker‐less with 6D couch (ML_6D).

Axis	Reference	Mean ± SD Position couch shift (mm)	Comparison	Mean ± SD Position couch shift (mm)	*β*	95% CI	*p*‐value
Vertical	M	2.9 ± 1.1	M_6D	2.5 ± 2.20	−0.40	−1.6 to 0.9	0.53
			ML	2.1 ± 0.9	−0.82	−1.7 to 0.1	0.08
			ML_6D	2.4 ± 1.2	−0.55	−1.4 to 0.3	0.21
	M_6D	2.5 ± 2.2	ML	2.1 ± 0.9	−0.42	−1.5 to 0.7	0.47
			ML_6D	2.4 ± 1.2	−0.15	−1.2 to 0.9	0.78
	ML	2.1 ± 0.9	ML_6D	2.4 ± 1.2	0.26	−0.4 to 0.9	0.42
Longitudinal	M	3.3 ± 1.3	M_6D	3.3 ± 1.5	0.06	−1.5 to 1.5	1.00
			ML	2.8 ± 1.3	−0.57	−1.6 to 0.6	0.36
			ML_6D	3.0 ± 1.5	−0.29	−1.3 to 0.8	0.63
	M_6D	3.3 ± 1.5	ML	2.8 ± 1.3	−0.51	−1.8 to 0.8	0.45
			ML_6D	3.0 ± 1.5	−0.25	−1.5 to 1.0	0.70
	ML	2.8 ± 1.3	ML_6D	3.0 ± 1.5	0.26	−0.5 to 1.0	0.51
Lateral	M	2.6 ± 1.6	M_6D	2.3 ± 1.6	−0.26	−1.4 to 0.9	0.65
			ML	1.6 ± 0.5	−1.01	−1.8 to ‐0.2	0.02^*^
			ML_6D	1.8 ± 1.1	−0.79	−1.6 to ‐0.0	0.06
	M_6D	2.3 ± 1.6	ML	1.6 ± 0.5	−0.74	−1.7 to 0.3	0.15
			ML_6D	1.8 ± 1.1	−0.53	−1.5 to 0.4	0.28
	ML	1.6 ± 0.5	ML_6D	1.8 ± 1.1	0.21	−0.4 to 0.8	0.49
Rotation	M_6D	1.2 ± 0.7	ML_6D	0.9 ± 0.4	−0.29	−0.7 to 0.2	0.19
Pitch		0.6 ± 0.3		0.9 ± 0.5	0.32	−0.1 to 0.8	0.14
Roll		0.9 ± 0.7		0.9 ± 0.5	−0.20	−0.7 to 0.3	0.42

* Statistically significant at *p* < 0.05.

β, regression coefficient, representing the mean difference between the comparison and reference groups; a negative β indicates a lower outcome in the comparison group.

For rotational shifts, the marker group exhibited mean rotations of 1.2 ± 0.7° (yaw), 0.6 ± 0.3° (pitch), and 0.9 ± 0.7° (roll). In comparison, the marker‐less group showed corresponding average rotations of 0.9 ± 0.4°, 0.9 ± 0.5°, and 0.7 ± 0.5°, respectively. No statistically significant differences in rotational movement were found between marker‐based and marker‐less setups when using a 6D couch. However, the marker‐less groups exhibited more consistent rotational variations across all axes.

### 3.3 Setup time

**FIGURE 8:**
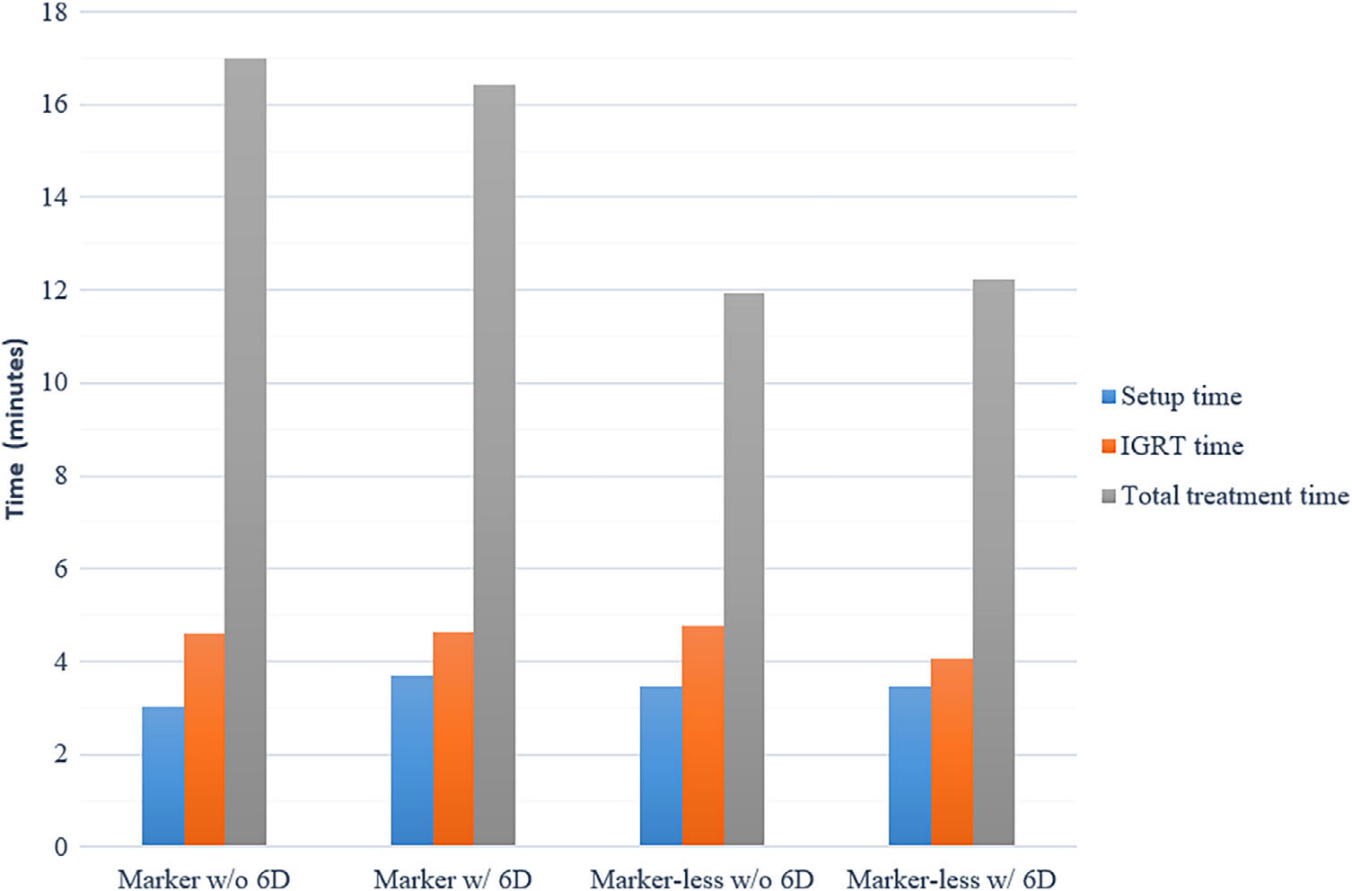
Comparison of average setup time, IGRT time, and total treatment time between groups with and without the use of a 6D couch. “w/” = with 6D couch; “w/o” = without 6D couch.

The average setup time, IGRT time, and total treatment time are illustrated in Figure 8. The average setup times were 3.0 ± 1.6, 3.7 ± 1.2, 3.5 ± 1.6, and 3.5 ± 1.3 min for the M, M_6D, ML, and ML_6D groups, respectively. The corresponding IGRT times were 4.6 ± 2.0, 4.6 ± 2.7, 4.8 ± 1.9, and 4.1 ± 1.6 min. No statistically significant differences were observed among the groups for either setup time or IGRT time. The GEE analysis results for average total treatment times are summarized in Table 2. When comparing setups with and without a 6D couch (M vs. M_6D and ML vs. ML_6D), no significant differences in total treatment time were found (*p* = 0.58 and *p* = 0.59, respectively). In contrast, the marker‐less groups demonstrated significantly shorter total treatment times compared to the marker groups.

**TABLE 2:** GEE analysis of total treatment time comparisons among four groups: Marker without 6D couch (M), marker with 6D couch (M_6D), marker‐less without 6D couch (ML), and marker‐less with 6D couch (ML_6D).

Reference	Mean ± SD total treatment time (minutes)	Comparison	Mean ± SD total treatment time (minutes)	β	95% CI	p‐value
M	17.0 ± 2.9	M_6D	16.4 ± 1.6	−0.58	−2.6 to 1.5	0.58
		ML	12.0 ± 2.1	−5.06	−6.6 to ‐3.6	<0.001^*^
		ML_6D	12.3 ± 1.9	−4.77	−6.2 to ‐3.4	<0.001^*^
M_6D	16.4 ± 1.6	ML	12.0 ± 2.1	−4.47	−6.3 to ‐2.6	<0.001^*^
		ML_6D	12.3 ± 1.9	−4.18	−5.9 to ‐2.4	<0.001^*^
ML	12.0 ± 2.1	ML_6D	12.3 ± 1.9	0.29	−0.8 to 1.4	0.59

* Statistically significant at *p* < 0.05.

β, regression coefficient, representing the mean difference between the comparison and reference groups; a negative β indicates a lower outcome in the comparison group.

## 4 DISCUSSION

This study investigated the emotional experiences associated with temporary skin marking among RTTs and breast cancer patients undergoing radiotherapy using a structured questionnaire. Approximately 90% of both patients and RTTs preferred a marker‐less setup. In addition, the accuracy of patient positioning using marker‐based and marker‐less techniques, with and without 6D couch correction, was evaluated based on couch shift values obtained from CBCT verification. The marker‐less approach combined with 6D couch correction demonstrated positioning accuracy comparable to the traditional marker‐based method while also reducing the overall treatment process time by 27.8%.

Temporary skin marking has previously been shown to induce psychological stress in breast irradiation patients,^2^, ^3^ and our finding suggests that this effect is particularly pronounced among the employed patients. Beyond emotional distress, skin marking imposes lifestyle limitations, including restrictions on showering, skincare use, and clothing choices to prevent fading. Asada et al.^2^ reported that 56% of patients undergoing radiotherapy felt uncomfortable with skin marking pens.

In our study, 90% of patients expressed a preference for a marker‐less setup. However, 10% of patients still accepted the use of skin marking. As discussed by Asada et al.,^2^ some patients understand the perceived necessity of skin markings for facilitating accurate treatment delivery.

The positioning accuracy observed in our study is consistent with findings from Sasaki et al.,^10^ with the largest deviations occurring in the longitudinal direction. No statistically significant differences in setup accuracy were identified between the marker and marker‐less groups, consistent with the observations reported by Jeong et al.^9^ and Sasaki et al.^10^ Rotational setup errors across all groups were within 2°, slightly lower than the 3° reported by Sasaki et al.^10^

These results indicate that the use of a 6D couch did not significantly affect setup accuracy in either the marker‐based or marker‐less groups. This finding may be attributable to differences in skin marking techniques. While Mueller et al.^11^ employed spot tattoos and Mast et al.^12^ recommended three‐point skin marks, our study used linear strip markings, which may have influenced alignment differently. The marker‐less group exhibited lower variability in positioning.

The average setup time in our study (∼3.5 min) was comparable to the 3.7 min reported by Jeong et al.^9^ Although setup and IGRT times were similar between groups, the marker‐less workflow reduced the total treatment time (4–5 min difference) by eliminating the need to re‐delineate faded skin marks and the time required for RTT and patient counseling on proper care of the temporary skin markings, as well as any additional instructions requested by the patient. This contributed to shorter overall treatment durations and reduced delays in room turnover.

Previous studies have compared the setup time of SGRT with traditional skin marker‐based approaches.^13^, ^14^, ^15^ In contrast, our study differentiated setup time, IGRT time, and total treatment time, allowing us to identify which components contributed most to overall treatment duration. In the marker group, frequent reapplication of faded skin markings lengthened the total treatment time relative to the marker‐less group.

In this study, time measurements were collected during the first three treatment fractions and weekly thereafter. A direct comparison of setup time between the first and subsequent fractions was beyond the scope of this study. However, Jeong et al.^9^ reported significantly reduced setup times using SGRT both on the first treatment day and across all fractions. Conversely, Svestad et al.^14^ found no significant differences between tattoo‐based and SGRT setups at different time points.

This study has several limitations. First, the comparison between marker‐based and marker‐less setups was performed on different patients, introducing potential inter‐individual variability. Second, the emotional responses reported by patients reflect their experiences with temporary skin markers. However, the study by Lastrucci et al.^16^ indicates that permanent tattoos may elicit stronger or qualitatively different emotional reactions. Third, the small sample size per group (*n* = 10 may limit statistical power to detect clinically meaningful differences. Additionally, the size and shape of the ROI can influence the registration accuracy of patient setup. AlignRT is known to be sensitive to surface topography landmarks,^17^ particularly in the breast region.^10^ In some cases, the contralateral breast may be unintentionally included in the ROI, potentially affecting the surface matching. Giantsoudi et al.^8^ addressed this concern by expanding the ROI to include a strip of skin below the contralateral breast extending to the mid‐axillary line, while excluding the contralateral breast to improve registration reliability.

## 5 CONCLUSION

Patients and staff demonstrated a clear preference for a marker‐less setup. SGRT proved effective for breast irradiation without the need for skin markings and may help reduce patient stress. Marker‐less setups provided positioning accuracy comparable to marker‐based methods, with no significant differences observed. The use of a 6D couch did not significantly affect setup accuracy or overall process time in either the marker‐based or marker‐less groups. These findings support the feasibility and clinical utility of SGRT as a setup technique for breast radiotherapy.

## AUTHOR CONTRIBUTIONS

**Puntiwa Oonsiri**: Conceptualization; methodology and writing—original draf. **Sornjarod Oonsiri**: Project administration and supervision. **Sakda Kingkaew**: Visualization; investigation and writing—review and editing. **Mananchaya Vimolnoch**: Visualization; investigation and writing—review and editing. **Jumnong Kumkhwao**: Data curation. **Photong Duangsuphan**: Data curation. **Panicha Nualsutha**: Data curation. **Nattawat Samranjai**: Data curation. **Siriporn Wong**: Data curation and resources. **Kitwadee Saksornchai**: Writing—reviewing and editing.

## CONFLICT OF INTEREST STATEMENT

The authors declare that they have no known competing financial interests or personal relationships that could have appeared to influence the work reported in this paper.

## ETHICS STATEMENT

This study was approved by the Institutional Review Board, Faculty of Medicine, Chulalongkorn University (IRB No. 0395/2025).

## Data Availability

All data generated or analyzed during this work are included in this published article.
